# Position of Fovea Palatinae relative to the vibrating line in various soft palate classifications among Jordanian edentulous population

**DOI:** 10.1038/s41598-024-60162-y

**Published:** 2024-04-26

**Authors:** Wijdan R. Elmanaseer, Rasha A. Alamoush, Firas Alsoleihat, Mahmoud K. AL-Omiri

**Affiliations:** 1 Department of Fixed and Removable Prosthodontics, School of Dentistry, University of Jordan, Amman, 11942 Jordan; 2 Department of Restorative Dentistry, School of Dentistry, University of Jordan, Amman, 11942 Jordan; 3 Department of Prosthodontics, The City of London Dental School, Canada Water, Lower Road, London, UK

**Keywords:** Fovea Palatinae, Posterior vibrating line, Soft palate angulation, House classification, Anatomy, Oral anatomy

## Abstract

This study aims to examine the relationship between the locations of Fovea Palatinae and the posterior vibrating line in different classes of soft palate angulation (House Classification), accordingly determine its reliability as a landmark and a tool for determining the posterior limit of the maxillary complete denture. 280 completely edentulous patients with normal healthy mucosa from both genders were randomly selected. The House classification of the soft palate angulation was identified and recorded as Class I, II, or III. Phonation was used to determine the position of the vibrating line. The Fovea Palatinae was then marked. Then, the distance between the Fovea Palatinae and the vibrating line was measured and recorded. Finally, the relative position of the Fovea Palatinae to the vibrating line was recorded as being anterior, posterior, or on the vibrating line. The Chi Square test, the effect size measures (Eta and Cramer’s V tests), The Spearman’s Rho rank correlation test, and multinominal logistic regression analysis were utilized to analyse the data. House classification percentages were measured among people whose Fovea Palatinae was detectable; Class II palate was the most prevalent (47.14%), followed by Class I (43.93%), and then Class III (8.93%). Based on vibrating line position, 129 (58%) had a vibrating line anterior to Fovea Palatinae, 57 (26%) on the Fovea Palatinae, 36 (16%) posterior to Fovea Palatinae, and in 58 (21%) Fovea Palatinae were not detected. The mean distance between the vibrating line and Fovea Palatinae was 3.66 ± 1.6 mm anteriorly and 2.97 ± 1.36 mm posteriorly. No significant differences were found between males and females in regard to House classification and vibrating line position. The odds of having the fovea posterior to the vibrating line would increase by 5% for each year increase in the age (*P* = 0.035, odds ratio = 1.050). Class II House classification of the soft palate was found to be the most prevalent among the study participants. Also, the vibrating line was anterior to the Fovea Palatinae in the majority of cases. The odds of having the fovea posterior to the vibrating line would increase by age. The Fovea Palatinae could be considered a useful guide for locating the vibrating line.

## Introduction

The position of the posterior border of upper complete denture is important for successful posterior palatal seal, and it ends at an imaginary vibrating line that demarcates the movable and non-movable parts of the soft palate^1^. The vibrating line might be identified utilizing various methods such as oral digital scans^2–4^, 3D computed tomography^5^, functional swallowing/impression methods^6^, conventional techniques using phonetics (saying ah), Valsalva maneuver, soft tissue palpation and/or anatomical/arbitrary identification depending on anatomy of the soft palate and the position of the fovea palatine^1,3,4,7–11^.

Similar denture retention was reported when conventional techniques and arbitrary techniques were used to identify the vibrating line and establish the posterior palatal seal^10^. The posterior palatal seal area was found to be among the worst fitting portions of the inner surface of upper complete dentures that were digitally fabricated using CAD-CAM systems and 3D printing^4^. However, other researchers found that digitally fabricated denture bases had better posterior palatal seal adaptation than conventionally fabricated dentures^12^. Nevertheless, the use of conventional methods is viable and suitable to identify the vibration line and establish the posterior palatal seal of upper complete dentures. To that end, visual observation is the main method for locating the vibrating line^7^. Moreover, using anatomical landmarks like Fovea Palatinae and Hamular notches is considered one of the easiest and most commonly used methods in dental practice^13^.

The contour and angulation of soft palate (House classification of soft palate) impact the position of the vibrating line and the posterior palatal seal^14,15^. Shorter anterior posterior soft palate and soft palate with larger angles are associated with narrower posterior palatal seal and shorter distance between anterior and posterior vibrating lines^14,15^. This highlights the importance of visual observation in identification of the vibrating line.

During their undergraduate training, dental students need to learn how to demarcate vibrating lines, especially the posterior vibrating line, and the techniques to determine the posterior border of the upper denture have been continuously evaluated and examined over the past years^1–4,7–11^. However, using anatomical landmarks like Fovea Palatinae and Hamular notches is still considered one of the easiest and most commonly used methods in dental practice^13^. However, the relative position of the vibration line and the Fovea is subjected to great variability, and the vibrating line could be located posterior, anterior, or on the Fovea itself^1,7,9,13^.

So far, no studies have been conducted on the Jordanian population to assess the relationship between intra-oral anatomical landmarks and upper denture extensions. The aims of this study are to examine the relationship between the locations of Fovea Palatinae and the posterior vibrating line in different House classifications of soft palate angulation, accordingly determine its reliability as a landmark and a tool in determining the posterior limit of the maxillary complete denture.

The aims of this study are to examine the relationship between the locations of Fovea Palatinae and the posterior vibrating line, and to identify variations in this relationship between different House classifications of soft palate angulation. In addition, to identify differences between genders in this regard.

The null hypothesis was that there is no difference in the position of fovea Palatinae in relation to the vibrating line, and that this relation is not affected by the angulation of soft palate. In addition, no difference is present between genders in this regard.

## Materials and methods

The study was conducted in the Department of Prosthodontics, Jordan University Hospital, Jordan, and was approved by the Institutional Review Board/Deanship of Scientific Research of the University of Jordan (Ref # 10/2020/10983). This cross-sectional, non-interventional, and observational study was carried out on 280 completely edentulous patients who sought complete denture treatment at the Department of Prosthodontics (213 of them are males (76.1%) and 67 are females (23.9%)) age ranged between 29 and 88 years old (mean = 59.44, SD = 10.21, 95% CI of mean = 58.24–60.64). A gender stratified simple random sampling using computer generated random numbers was followed during the recruitment of the participants who visited the department seeking complete denture treatment. To be included, the participants should be completely edentulous, new to dentures, have normal healthy mucosa at the hard and soft palate, and have no medical condition that affects mucosa or muscles such as Parkinson’s disease, velopharyngeal dysfunction, vesiculobullous disease, and neurological disorders. Participants with palatal tori, maxillary tuberosity undercuts, or other abnormalities, anomalies, or defects that affect the hard and soft palate were excluded from the study. Participation in this study was voluntary, and all participants provided their written informed consent before being included.

Two experienced observers (prosthodontist and well-trained dentist) examined and filled out the study sheets. Firstly, all the steps involved in the examination procedure were explained to the participants. Then, the participants were examined using a dental oral mirror (15/16 inch, Hanhnenkratt GMBH, Germany) on a dental chair with an illumination unit (Daray Lighting Ltd, Leighton Buzzard, and Bedfordshire, UK). The participants were requested to open their mouths widely with their heads in an upright position for better visualization and inspection of the soft and hard palate for any abnormality and for the presence of Fovea Palatinae.

**Figure 1:**
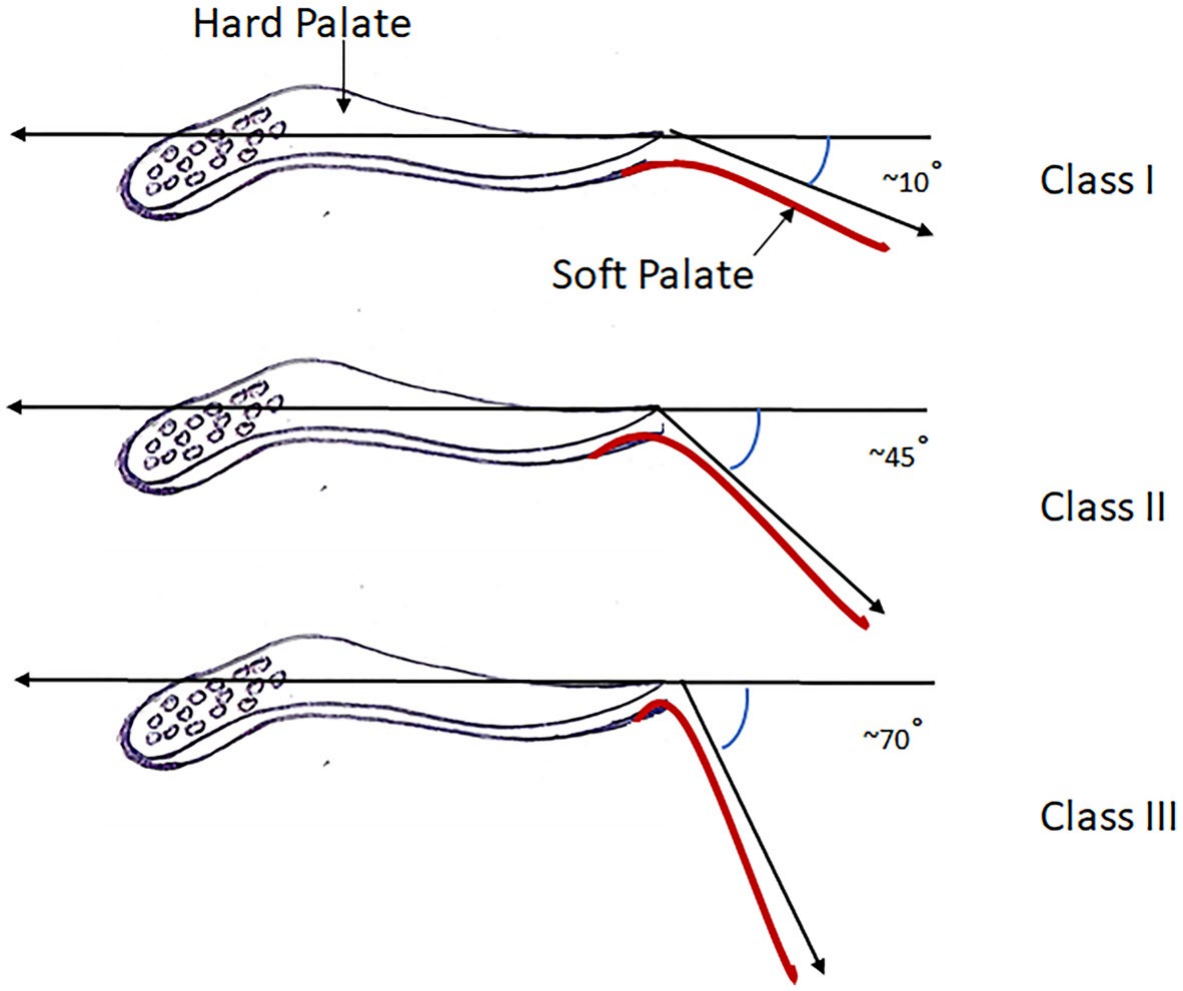
House Classification of soft palate, the angle formed between the soft palate and the hard palate determine the classification into type I, II, and III.

Then, the House classification of the soft palate angulation was identified by sitting the participants in the dental chair while their heads in an upright position. Then, the participants were requested to insert the index and middle fingers between their upper and lower arches in order to obtain a normal mouth opening of about 20 mms. While the mouth was open, the investigator inspected and visualized the position of the soft palate in relation to the hard palate. The angle between the antero-posterior extension of hard palate and the curved soft palate was observed and visually estimated then subsequently validated with a caliper. The House classification was then recorded as Class I (soft palate is horizontal or with an angle up to 10° with the hard palate), II (soft palate is moderately steep at an angle of 45° with the hard palate), or III (soft palate is steep at an angle of around 70° with the hard palate) following Millsap^16^ (Fig. 1).

**Figure 2:**
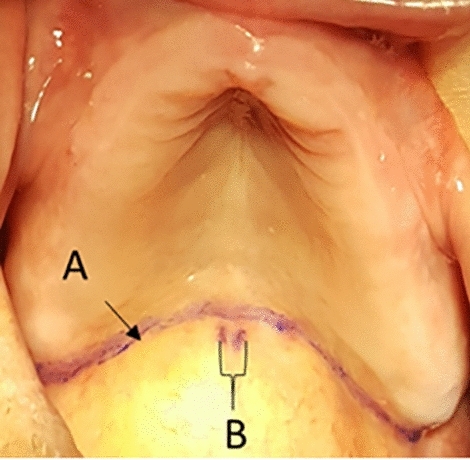
A clinical image of and an edentulous upper arch, the vibrating line (**A**) and Fovea Palatinae (**B**) drawn using indelible pencil.

After that, the soft and hard palate was dried using 2 × 2 cm gauze, this step not only aids in eliminating any mucus and saliva that may obscure the soft palate but also facilitates clearer visualization of the fovea palatinae. Consequently, this enhances the precision of drawing the vibrating line with an indelible pencil. The participants were instructed to say the “Ah” sound slowly in short bursts for five times in a normal unexaggerated fashion, in order to practice the “Ah” sound for vibrating line. While the participants were saying “Ah”, the soft palate was inspected to demarcate the position of the vibrating line, and using an indelible pencil the vibrating line was drawn on the participants' soft palate, starting from the Hamular notches and sliding towards the middle. Then, the Fovea Palatinae was marked using the same pencil (Fig. 2). After that, while the participant’s mouth was still open, they were instructed again to say “Ah” slowly to double-check the correct position of the vibrating line. Then, using a single-ended colour-coded periodontal probe with 1mm unit graduations and measurement accuracy (UNC15 anatomical handle periodontal probe, ASA Dental Co, Italy), the distance between the Fovea Palatinae and the vibrating line was measured and recorded on the examination sheet.

To control for measurement error and ensure measurements accuracy and consistency, all measurements started from the Fovea Palatinae and ended at the vibrating line. Also, each measurement was repeated twice, and an average of the two measurements was recorded as the final measurement value. In addition, all measurements were recorded by one investigator (H.S.). Finally, the relative position of the Fovea Palatinae to the vibrating line was recorded as being anterior, posterior, or on the vibrating line. The method error was tested by assessing the inter-examiner and intra-examiner reliability of the measurements of the distance between the Fovea Palatinae and the vibrating line. This was evaluated by repeating the distance measurement for 10 participants on two different occasions with one week interval by the same investigator (intra-examiner reliability) and by another investigator (inter-examiner reliability); then the agreements between the measurements were assessed utilizing kappa statistics. Kappa **(κ)** value was adequate and ranged between 0.92 and 0.95 indicating adequate reliability of the measurements.

### Statistical analysis

After data collection, Statistical software (IBM SPSS Statistics v19.0; IBM Corp., USA) was used for analysis. In this study, the categorical variables were presented as frequencies and percentages, while the continuous variables were presented as means and medians.

The Chi Square test and the effect size measures (Eta and Cramer’s V tests) were used to identify the associations between House classification, position of the Fovea Palatinae in relation to the vibrating line, gender, and age. The Spearman’s Rho rank correlation test was used to test the correlations between age and the distance between the Fovea Palatinae and the vibrating line.

Multinominal logistic regression analysis was used to predict the position of Fovea Platinae in relation to the vibrating line based on House classification, gender, and age. Also, hierarchical and backward multiple linear regression analyses were used to identify the distance between Fovea Palatinae and the vibrating line based on House classification, gender, and age. The confounding effects of gender and age were taken into consideration during the regression analyses.

Significant statistical analysis was detected at 0.05 two tailed probability value and 95% confidence intervals throughout this study.

The sample size calculation was carried out via priori power analysis utilizing the G*Power software (version 3.1.9.7; Heinrich-Heine University). With an α of 0.05, an odds ratio of 1.5, and a study power of 0.8; a logistic regression test estimated a minimum required sample of 242 participants. Two hundred and eighty participants were recruited to meet the required sample size throughout the study. None of the recruited participants was excluded or dropped out of the study after inclusion.

### Ethical approval

This was approved by the Institutional Review Board/Deanship of Scientific Research of the University of Jordan (Ref # 10/2020/10983). A written consent was obtained from each participant prior participation.

## Results

In this study, 280 completely edentulous patients (males 213 (76.1%) and females 67 (23.9%)) were examined. Participants’ age ranged between 29 and 88 years old (mean = 59.44, SD = 10.21, 95% CI of mean = 58.24–60.64, Median = 60.00, Interquartile range = 13).

Table 1 presents the descriptive statistics and the distribution of gender, House classification of soft palate, and position of the Fovea Palatinae in relation to the vibrating line among the participants in this study. House classification percentages were measured among people whose Fovea Palatinae was detectable; Class II palate was the most prevalent (47.14%), followed by Class I (43.93%), and then Class III (8.93%). Also, the Fovea Palatinae was not clinically detected in 20.36% of the participants. Among the participants who had their Fovea Palatinae detected, the Fovea Palatinae was detected anterior to the vibrating line (57.85%), on the vibrating line (26.01%), or posterior to the vibrating line (16.14%).

**Table 1:** Descriptive statistics and the distribution of gender, House classification of soft palate, and position of the Fovea Palatinae in relation to the vibrating line among the study population.

Variable	Descriptive statistics	
	n	%
Gender (n = 280)		
Males	213	76.07
Females	67	23.93
House Classification (n = 280)		
Class I	123	43.93
Class II	132	47.14
Class III	25	8.93
Detected Fovea Palatinae (n = 280)		
No	57	20.36
Yes	223	79.64
Position of Fovea Palatinae in relation to vibrating line (n = 222)		
Anterior to vibrating line	129	57.85
On the vibrating line	58	26.01
Posterior to vibrating line	36	16.14

Table 2 demonstrates the distribution of the position of the Fovea Palatinae in relation to the House classification of the soft palate among the study population. No significant relationships were detected between the position of the Fovea Palatinae and the House classification of the soft palate (Chi Square = 8.193, df = 4, P = 0.085, Cramer’s V = 0.136, Table 2).

**Table 2:** The distribution of the position of the Fovea Palatinae in relation to the House classification of the soft palate among the study participants.

	House classification of soft palate			Chi Square test			
	I	II	III	X^2^	df	P	Cramer’s V
Fovea Palatinae detected clinically (n = 280)							
No (n = 57)	23	26	8	2.334	2	.311	.091
Yes (n = 223)	100	106	17				
Fovea Palatinae position (n = 223)							
Anterior to vibrating line (n = 129)	65	54	10	8.193	4	.085	.136
On vibrating line (n = 58)	21	35	2				
Posterior to vibrating line (n = 36)	14	17	5				

*df* Degree of freedom, *X*^*2*^ Chi Square statistic, *P* Probability value using Chi Square test, *Cramer’s V* Measure of effect size.

The findings of the study showed that when the Fovea Palatinae was anterior to the vibrating line, the distance between the Fovea Palatinae and the vibrating line ranged between 1 and 8 mm (mean distance = 3.57 mm, SD = 1.64, SE of mean = 0.145, 95% CI of mean = 3.28–3.85, Median = 3.00, Interquartile range = 3). Meanwhile, when the Fovea Palatinae was posterior to the vibrating line, the distance between the Fovea Palatinae and the vibrating line ranged between 1 and 7 mm (mean distance = 2.97 mm, SD = 1.36, SE of mean = 0.227, 95% CI of mean = 2.51–3.43, Median = 3.00, Interquartile range = 2).

Table 3 shows the descriptive statistics of the distance between the Fovea Palatinae and the vibrating line considering the House classification of soft palate. No relationship was found between House classification and the distance between Fovea Palatinae and vibrating line when the Fovea Palatinae was located anterior to the vibrating line (Chi Square = 21.471, df = 14, *P* = 0.090, Table 3). However, Class II soft palate was associated with wider distance between the Fovea Palatinae and the vibrating line when the Fovea Palatinae was located posterior to the vibrating line (Chi Square = 21.863, df = 12, *P* = 0.039, Table 3).

**Table 3:** the descriptive statistics of the distance between the Fovea Palatinae and the vibrating line considering the House classification of soft palate among the participants.

Distance between Fovea Palatinae and vibrating line	Descriptive statistics	House classification			Chi Square test		
		Class I	Class II	Class III	X^2^ (df)	P	Eta
When Fovea Palatinae anterior to vibrating line	Mean (mm)	3.662	3.370	4.000	21.471 (14)	0.090	0.115
	SE	0.2025	0.2216	0.5774			
	SD	1.630	1.629	1.826			
	95% CI	3.257–4.066	2.926–3.815	2.694–5.306			
	Median	3.00	3.00	4.50			
	IQR	2.5	2.0	2.8			
	Minimum	1	1	1			
	Maximum	8	7	6			
When Fovea Palatinae posterior to vibrating line	Mean (mm)	2.857	3.176	2.600	21.863 (12)	0.039	0.156
	SE	0.3120	0.2743	1.1225			
	SD	1.167	1.131	2.510			
	95% CI	2.183–3.531	2.595–3.758	0.517–5.717			
	Median	3.00	3.00	2.00			
	IQR	1.0	1.5	3.5			
	Minimum	1	1	1			
	Maximum	6	5	7			

*SE* Standard error, *SD* Standard deviation, *95% CI* 95% Confidence intervals, *IQR* Interquartile range, *df* Degree of freedom, *X*^*2*^ Chi Square statistic, *P* Probability value using Chi Square test, *Eta* Measure of effect size considering the distance as the dependent variable.

Significant relationships were identified between age and House classification of soft palate. Younger age was associated with presence of Class I soft palate while older age was associated with Class III soft palate (Chi Square = 133.845, df = 90, *P* = 0.002, Eta = 0.527, effect size = 0.2777). However, no significant relationship was identified between age and the position of Fovea Palatinae in relation to the vibrating line (Chi Square = 107.360, df = 88, *P* = 0.079, Eta = 0.537, effect size = 0.2884). Also, no significant correlation was found between age and the distance between Fovea Palatinae and vibrating line when the Fovea Palatinae was anterior (Sperman’s rho = 0.057, *P* = 0.057) or posterior (Sperman’s rho = 0.001, *P* = 0.994) to the vibrating line.

Also, no significant differences between genders were found regarding the House classification of soft palate (Chi Square = 2.551, df = 2, *P* = 0.279, Cramer’s V = 0.095), the position of Fovea Palatinae in relation to the vibrating line (Chi Square = 0.984, df = 2, *P* = 0.612, Cramer’s V = 0.066), nor the distance between Fovea Palatinae and the vibrating line when the Fovea Palatinae was anterior (Chi Square = 8.070, df = 7, *P* = 0.326, Eta = 0.158, effect size = 0.0250) or posterior (Chi Square = 3.974, df = 6, *P* = 0.680, Eta = 0.059, effect size = 0.0035) to the vibrating line.

Multinominal logistic regression analyses (Table 4) were conducted to identify the odds of the position of Fovea Palatinae in relation to vibrating line based on House classification, gender, and age. The results revealed that in comparison to having the Fovea Palatinae on the vibrating line, the odds of having the fovea posterior to the vibrating line will increase by 5% for each year increase in the age of the participant (Nagelkerke R^2^ = 0.069, B = 0.049, SE = 0.023, Wald = 4.459, df = 1, *P* = 0.035, odds ratio = 1.050, 95% of the odds ratio = 1.003–1.098). Also, linear multiple regression analyses (both hierarchical and backward methods) were conducted to predict the distance between the Fovea Palatinae and the vibrating line; and none of the models or the factors was significant in this regard.

**Table 4:** Multinominal regression analysis to predict the position of the Fovea Palatinae in relation to the vibrating line among the study population.

FP Position	Predictors	B	SE	df	*P*	Odds ratio	95% CI for OR	
							Lower Bound	Upper Bound
FP Anterior to VL compared to FP on VL (R^2^ = 0.069)	Intercept	0.204	1.414	1	0.885	–	–	–
	Age	0.016	0.017	1	0.345	1.016	0.983	1.050
	Gender	0.278	0.386	1	0.471	1.321	0.620	2.813
	House class I	− 0.315	0.829	1	0.704	0.730	0.144	3.710
	House class II	− 1.068	0.810	1	0.187	0.344	0.070	1.682
	House class III	0	–	0	–	–	–	–
FP Posterior to VL compared to FP on VL (R^2^ = 0.069)	Intercept	− 2.717	1.863	1	0.145	–	–	–
	Age	0.049	0.023	1	0.035	1.050	1.003	1.098
	Gender	0.306	0.503	1	0.542	1.358	0.507	3.638
	House class I	− 0.868	0.935	1	0.353	0.420	0.067	2.622
	House class II	− 1.368	0.904	1	0.130	0.255	0.043	1.496
	House class III	0	–	0	–	–	–	–
FP Posterior to VL compared to FP Anterior to VL (R^2^ = 0.069)	Intercept	− 2.921	1.586	1	0.065	–	–	–
	Age	0.033	0.020	1	0.104	1.033	0.993	1.074
	Gender	0.028	0.427	1	0.948	1.028	0.445	2.377
	House class I	− 0.553	0.652	1	0.396	0.575	0.160	2.064
	House class II	− 0.300	0.630	1	0.634	0.741	0.216	2.544
	House class III	0	–	0	–	–	–	–

*FP* Fovea Palatinae, *VL* Vibrating line, *R*^*2*^ Nagelkerke Coefficient of determination, *B* Beta statistics, *SE* Standard Error, *df* Degree of freedom, *P* Two tailed probability value, *CI for OR* Confidence intervals for the odds ratio.

## Discussion

The precise positioning of the posterior border of the upper complete denture is critical for establishing an effective posterior palatal seal. This border terminates at an imaginary line known as the vibrating line, which delineates the boundary between the movable and immovable portions of the soft palate.

The outcomes of this investigation demonstrated that the Fovea Palatinae has various locations in relation to the posterior vibrating line, and that this relationship is different in different House classifications of soft palate angulation. Therefore, the null hypothesis was rejected considering these assumptions. However, no differences were found between genders, and thus the null hypothesis was accepted in this regard.

In this investigation, the posterior vibrating line was utilized during the measurements because it is considered the marking point for the posterior extension of maxillary prostheses in most literature^1,6,9,10,17–19^.

Phonation (saying Ah) and anatomical landmark (Fovea Palatinae) were utilized in this study to identify the position of the posterior vibrating line because anatomical landmarks would provide an easy guide to locate the vibrating line, and this has been considered practical and clinically viable by previous researchers^1,6,9,10,13,16–20^.

The findings of this study showed that the fovea Palatinae was not detected in 21% of the participants. This compares to previous studies that could not detect the Fovea Palatinae in around 27% of the examined participants^18^.

Among those who had their Fovea Palatinae detected in this study, 58% had the vibrating line anterior to the Fovea Palatinae (mean distance = 3.66 ± 1.6 mm), 26% on the Fovea Palatinae, and 16% posterior to the Fovea Palatinae (mean distance = 2.97 ± 1.36 mm). In comparison, previous studies reported the vibrating line to be anterior to the Fovea Palatinae in 80%^21^, 75%^22^, 8.6%^18^, and 45%^17^ of the participants. Also, the vibrating line was on the Fovea Palatinae in 20%^21^, 25%, 32.5%^18^, and 50.6%^17^ of the cases. In addition, the vibrating line was reported to be posterior to the Fovea Palatinae in 0%^21,22^, 31.1%^18^ and 4.4%^17^ of the participants. Although there are some variations regarding the vibrating line position in relation to the Fovea Palatinae, the vibrating line was mainly located anterior to or on the Fovea Palatinae in most previous studies^17,18,21,22^. This variation between the reported percentages of the vibrating line might be related to different races or ethnicity^14,15,21^, which might be a good area for research in the future.

Furthermore, this study reported larger distance between the Fovea Palatinae and the vibrating line when the vibrating line was anterior to the Fovea Palatinae (mean distance = 3.66 ± 1.6 mm) in comparison to previous studies (mean distance = 2.62 ± 1.68 mm^21^ and 2.58 ± 1.19 mm^9^). Similarly, this study reported larger distance between the Fovea Palatinae and the vibrating line when the vibrating line was posterior to the Fovea Palatinae (mean distance = 2.97 ± 1.36 mm) in comparison to a previous report by Kyung et al. (mean distance = 0.71 ± 0.68 mm)^9^. Again, this contrast from previous findings might be related to different races or ethnicity, which might be a good area for future investigations.

In this study, class II House classification of the soft palate was found to be the most prevalent (47.14%) among the participants. This agrees with the findings of Chaturvedi et al.^2^ who reported class II soft palate among 44% of participants from similar age group in southern Saudi province. This might be due to the demographic characteristics of the participants who were not very young or old.

The findings of this study showed that younger age was associated with the presence of Class I soft palate while older age was associated with Class III soft palate. This was substantiated by the finding that the odds of having the fovea posterior to the vibrating line would increase by 5% for each year increase in the age of the participant in comparison to having the Fovea Palatinae on the vibrating line. This finding might be explained by the potential impacts of aging on palatal muscles, palatine aponeurosis and soft palate structures leading to more steep contours of the soft palate^15,23^. This conforms to the findings of Kotlarek et al.^24^ who found that velar morphology and shape varied significantly with age. In contrast, age was found to have no effect on the type of soft palate among a southern Saudi population^2^. Also, age had no relationship with the position of Fovea palatinae in relation to the vibrating line or with the distance between the Fovea and the vibrating line. This reflects the long-term stability of these anatomical landmarks which make them viable to use for detection of the posterior border of upper dentures.

In this study, no statistical difference was found between male and female groups in terms of both House classification and vibrating line position. This agrees with the previous findings that found that velar morphology and shape was not affected by the sex of the participants^24^. However, this contrasts with other findings that reported higher soft palate angulations among females who were living in a southern Saudi province^2^. This variation might be due to variations in race, genetics and ethnicity^14,15,21^.

In this investigation, no relationship was found between House classification of the palate and the distance between Fovea Palatinae and vibrating line when the Fovea Palatinae was located anterior to the vibrating line. However, Class II soft palate was associated with wider distance between the Fovea Palatinae and the vibrating line when the Fovea Palatinae was located posterior to the vibrating line. This finding agrees with previous findings that lower palatal contours were associated with wider distance between Fovea Palatinae and the posterior vibrating line^9,21^. This might be explained by that less contoured soft palate would have wider area of non-mobile soft tissues that provide suitable bases for posterior palatal seal.

This study demonstrates the first report into the position of Fovea Palatinae in relation to vibrating line in various types of soft palate (various soft palate classifications) among Jordanian edentulous population. It provides areas for comparison to data from different populations with various ethnic, genetic, racial, and demographic backgrounds. The location of the vibrating line was mostly anterior to Fovea Palatinae in all House classifications. Hence, the Fovea Palatinae might be a useful guide in locating the vibrating line. Nevertheless, as Fovea Palatinae had a variable position or may be absent, it could be augmented with other methods to identify the posterior palatal seal area.

A potential limitation of the present study is depending on the investigators to identify the Fovea Palatinae, vibrating line, and the soft palate contour classification. However, highly experienced and well-trained professionals carried out the experiment and tested the inter- and intra- examiner reliability during the investigation. Also, clinical evaluation of the study parameters was considered viable by many professionals and researchers in the field. Another limitation is inability to observe the longitudinal effects of age on fovea palatinae position by following patients over time hence all the participants were new to dentures, additionally, exclude participants with jaw deformities. Nevertheless, this presents an area for future research.

It is recommended to conduct further investigations and comparative studies among other populations with different ethnic, racial and demographic backgrounds on larger samples in this regard.

## Conclusion

Within the limitations of this study, it was concluded that class II House classification of the soft palate was found to be the most prevalent among the study participants, and was associated with wider distance between the Fovea Palatinae and the vibrating line when the Fovea Palatinae was located posterior to the vibrating line. Also, the vibrating line was anterior to the Fovea Palatinae in the majority of cases.

In this study, the odds of having the fovea posterior to the vibrating line would increase by age. However, no significant difference was found between males and females in terms of both House classification of soft palate and the vibrating line position.

## Data Availability

The datasets used and/or analysed during the current study available from the corresponding author on reasonable request. Paper based forms were used to fill information from patients, then they were transferred to excel sheets. Statistical analysis was done using statistical software (IBM SPSS Statistics v19.0; IBM Corp., USA).
